# HISTORICAL NARRATIVES, CONTEMPORARY PORTRAYALS, AND STRATEGIES TO REFRAME AGING

**DOI:** 10.1093/geroni/igae098.0836

**Published:** 2024-12-31

**Authors:** Reuben Ng

**Affiliations:** National University of Singapore, Singapore, Singapore

## Abstract

Ageism is the discrimination of older adults based on their age, and the negative portrayals of older adults as derived from age-based stereotypes centered on illness, irrelevance, and incompetence. The insidious consequences of ageism to health prompted 194 member countries to collectively work through the World Health Organization on a global campaign to combat ageism. This talk showcases innovative methods in analyzing the historical narratives of aging over 200 years, contemporary media portrayals of older adults, and strategies to reframe aging.

